# Adsorption of Cadmium, Manganese and Lead Ions from Aqueous Solutions Using Spent Coffee Grounds and Biochar Produced by Its Pyrolysis in the Fluidized Bed Reactor

**DOI:** 10.3390/ma13122782

**Published:** 2020-06-20

**Authors:** Jarosław Chwastowski, Dariusz Bradło, Witold Żukowski

**Affiliations:** Department of Inorganic Chemistry and Technology, Cracow University of Technology, Warszawska 24, 31-155 Cracow, Poland; dariusz.bradlo@pk.edu.pl (D.B.); witold.zukowski@pk.edu.pl (W.Ż.)

**Keywords:** adsorption, cadmium, manganese, lead, biosorbents

## Abstract

The adsorption process of cadmium ions (Cd), manganese ions (Mn) and lead ions (Pb) onto the spent coffee grounds (SCG) and activated spent coffee grounds (biochar, A-SCG) was investigated. The SCG activation was carried out in the pyrolysis process in a fluidized bed reactor. scanning electron microscope (SEM) with energy dispersive X-ray spectroscopy (EDX), Fourier-transform infrared spectroscopy (FTIR), Brunauer–Emmett–Teller (BET) measurements and CHN analysis were used in order to define the differences between biomaterials. In the study the different mass of materials (0.2–0.5 g) and constant heavy metal volume and concentration (20 cm3/100 ppm) were investigated on the adsorption process. In order to describe the sorption parameters the Langmuir, Freundlich and Temkin isotherms were used. The maximum adsorption for biochar reached 22.3 mg/g for Pb ions, 19.6 mg/g for Mn ions and 19.4 mg/g for Cd ions which were noticeably higher than the results obtained for spent coffee grounds which reached 13.6 mg/g for Pb ions, 13.0 mg/g for Mn ions and 11.0 mg/g for Cd ions. Metal ion adsorption on both SCG and A-SCG was best described by the Langmuir model, thus chemisorption was a dominant type of adsorption. Studying the kinetics of the sorption process, one can see that the process is of a chemical nature according to the best fit of the pseudo-second rate order model. The obtained results show that the chosen sorbents can be used for the removal of cadmium, manganese and lead compounds from aqueous solutions with high efficiency.

## 1. Introduction

Increasing knowledge about heavy metal toxicity and legal requirements for industrial emissions reduction has led scientists to intensify R&D activity in the area of wastewater treatment [1]. Various aqueous solutions of heavy metal ions are used in industries such as metal processing, electroplating, tanning, etc. Accumulation of toxic compounds in various organisms has brought about the point where water remediation processes are extremely important. Biosorption is a cost-effective option for the removal of toxic metal compounds from aqueous solutions [2]. The use of biological materials including living and non-living organisms in the processes of removal and remediation of toxic metals has gained crucial credibility in recent years due to their good performance, ease of use and universal access [3].

Cadmium is a highly toxic metal that can cause lethal damage to various parts of the human body the bones and kidneys. It can cause erythrocyte destruction, diarrhea, nausea, salivation, muscular cramps, renal degradation and chronic pulmonary problems. The major industries that releases cadmium compounds into the environment are in the manufacturing of alloys, batteries, pigments and plastics. The WHO (World Health Organization) and AWWA (American Water Works Association) recommends that the drinking water should not exceed 0.005 mg/dm^3^ of Cd(II) [4].

Manganese can cause organoleptic and operating problems when present in groundwater. It can consume chlorine in the disinfection process and promote biofouling and corrosion in water networks caused by microorganisms [5]. Nowadays, there are various methods to remove Mn such as initial aeration followed by rapid filtration, pH adjustment and secondary rapid filtration or biofiltration with the use of manganese oxidizing bacteria that colonize the sorption bed. In nature the bacteria present in raw waters are able to proliferate in the sand filters under the optimal conditions and are able to oxidize the divalent manganese Mn(II) to their oxidized form Mn(IV) [6].

Lead is a hazardous heavy metal that is present in industrial wastewater. It causes diseases like mental disorder, anorexia or even death. Lead ions can be found in the process of refining ores, metal processing, sludge disposal, production of pesticides, metallurgical engineering and oxidation [7,8]. The most common ways to purify wastewater are ion exchange, precipitation and sorption processes. These processes are in general expensive and ineffective, especially when the concentrations of metals are in the range between 50–100 mg/cm^3^ [9].

Recent studies have shown that biochar has been used with success in metal-polluted water treatment due to its highly specific properties like hydrophobicity, surface charge, and surface area [10,11,12]. Naeem et al. used raw and acid activated wheat straw biochar for the removal of Cd ions from water with sorption capacities ranging between 31.65 mg/g and 74.63 mg/g [11]. Biochar is a black solid char derived from the pyrolysis of organic waste materials in a limiting oxygen environment [13]. It can be produced from spent coffee grounds in different types of equipment (e.g., a furnace [14,15], a tubular reactor [16,17] or a screw-conveyor reactor [18]). Each of these solutions has its own drawbacks, but the common feature of all is the limited mass and energy transfer, thus the temperature gradient can be observed as well as the disrupted diffusion of gases (into and from the sample). Novelty in the process of obtaining the biochar is to omit these difficulties through the use of the thermal process organized in the fluidized bed reactor. Bok et al. [19] performed fast pyrolysis of coffee grounds in a fluidized bed reactor where the bed was sand and the temperature was constant in the range of 400–600 °C. The material was dosed gradually and the flow of N_2_ was sufficient to ensure about 1 s residence time. Nevertheless, there have been no studies on the biochar produced from spent coffee grounds that consisted up to 100% by weight of the bed and the fluidization process was performed under the CO_2_ condition with long residence time.

The aim of this work was to study the difference between the spent coffee grounds (SCG) and activated spent coffee grounds (biochar, A-SCG) made through the thermal processing of SCG under specific conditions on its sorption properties for the removal of the three metal ions of cadmium, manganese and lead from aqueous solutions. The results of the metal sorption were evaluated through different isotherm and kinetic model studies. The practical aspect of the work is to use the waste material and turn it into high efficiency, renewable material for the removal of heavy metal ions from the wastewaters. Initial experiments, which concerned the spent coffee grounds, showed that in contact with water, it introduces organic compounds into the solution. This is clearly evidenced by a change in the color of the entire solution due to the biological nature of the raw material tested. From the point of view of wastewater treatment, this fact is unfavourable as it could lead to a situation in which the wastewater would be treated for one impurity (e.g., metal cations) and would be contaminated with other substances (e.g., organic compounds). Therefore, it was assumed that the stage of thermal transformation of the material is necessary, because if used on a larger scale, there is a risk of water pollution through sorbent decomposition processes.

## 2. Materials and Methods

### 2.1. Materials

One hundred percent Arabica coffee used in the studies was brewed at 100 °C and then washed with distilled water three times to remove the residues created during the brewing process. As received spent coffee grounds underwent further analysis. The A-SCG was made from the same spent coffee grounds during the process of pyrolysis. First, SCG was dried in 120 °C for 12 h, then screened in order to obtain particles in the range of 0.2–0.4 mm. Afterward the prepared material was again dried in 120 °C overnight. All the chemicals used in the study were of analytical grade from Sigma-Aldrich (Steinheim, Germany). The stock solutions of Cd(II), Mn(II) and Pb(II) (100 mg/dm^3^) were prepared by dissolving the appropriate amounts of cadmium sulfate(VI), manganese(II) sulfate(VI) and lead(II) nitrate(V), respectively, in 250 cm^3^ of demineralized water.

### 2.2. Methods

Surface analysis was done with scanning electron microscope (SEM) equipped with an energy-dispersive X-ray spectroscopy microanalyzer (EDS). Optical photographs were obtained by means of a TPL Trino stereoscopic microscope (Rhede, Germany) equipped with a DLT-Cam PRO 5 MP camera (Mińsk Mazowiecki, Poland). The elemental analysis was carried out with the use of a Perkin Elmer CHN analyzer type 2400 (Waltham, MA, USA). To specify the moisture and ash content in the samples, additional analysis was supplemented. In order to define the characteristic chemical bonds present in the sorbent, materials were subjected to Fourier transform infrared (FTIR) spectroscopy before and after the pyrolysis process. The research was performed using a Nicolet 380 Spectrometer (Thermo Fisher Scientific, Waltham, MA, USA). Surface analysis was carried out with the use of Macrometrics ASAP 2010 (Norcross, GA, USA) with a degassing station. First, the samples were dried at 110 °C under (He) conditions for 8 h and then at 100 °C in a vacuum of 0.001 Torr for 8 h. Metal content analysis was performed using inductively coupled plasma with optical emission spectrometry (ICP-OES) in a Perkin Elmer OPTIMA 7300 DV apparatus (Waltham, MA, USA).

**Figure 1:**
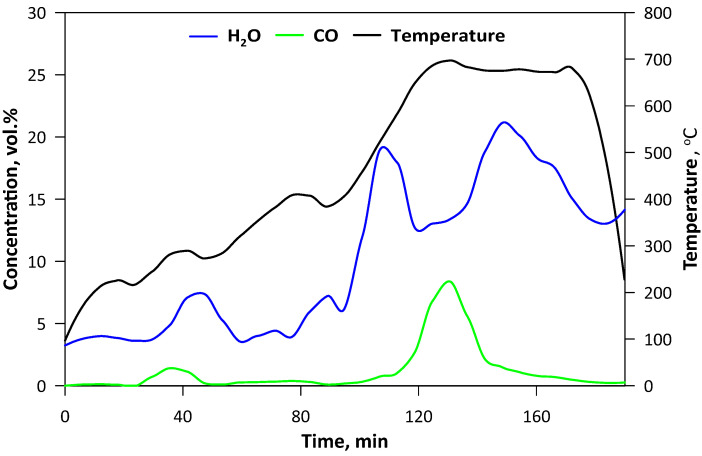
Temperature changes and contents of H_2_O and CO in the gaseous products during pyrolysis.

**Figure 2:**
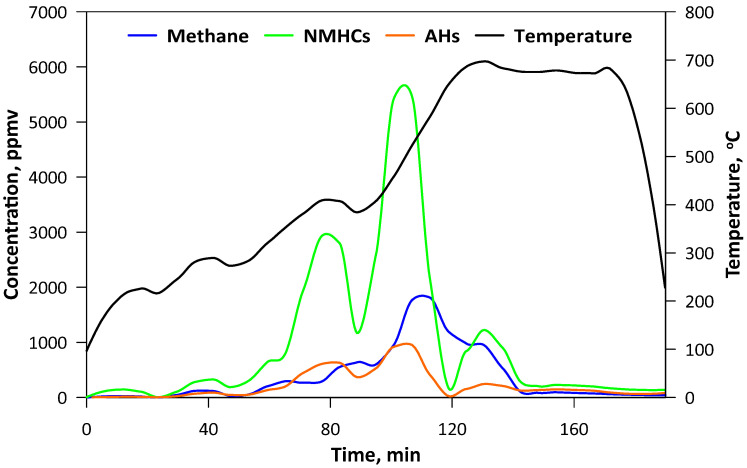
Concentration of hydrocarbons in the gaseous products of pyrolysis (NMHCs-nonmethane hydrocarbons; AHs-aromatic hydrocarbons).

The pyrolysis was carried out in the laboratory fluidized bed reactor that has been described previously in detail [20]. A total of 200 g of SCG was used as a bed. A quartz tubular reactor with an external diameter of 100 mm and height of 500 mm was equipped with an electrical heating jacket and insulation in order to maintain the desired temperature of the bed. To ensure pyrolytic conditions, CO_2_ as the fluidizing medium was applied. Its flow was controlled by a TSI 40241 flowmeter (Beijing, China) and the temperature was measured by the thermocouple located 50 mm above the perforated distributor. Gaseous products were monitored on-line by FTIR analyzer, Gasmet DX-4000 (Vantaa, Finland). Spectra of the gases were recorded at the intervals of 5 s, giving the information about the concentration of the major compounds (H_2_O, CO) as well as both organic (hydrocarbons, aldehydes, alcohols, esters etc.) and inorganic (nitric oxides, sulfur dioxide, ammonia, hydrochloric acid, cyanuric acid etc.) ones. The second analyzer (Horiba PG250, Kyoto, Japan) was used for measuring O_2_ (by electrochemical detector) and for comparative purposes NOx (by chemiluminescence method) with CO, CO_2_ and SO_2_ (by the nondispersive infrared detector, NDIR). Gaseous products of the process were transferred by the heating line (180 °C), however, due to intense liquid pyrolytic product condensation, an additional series of water scrubbers were used before the analytical apparatus. Thus, the concentration of H_2_O may be influenced by its condensation in the scrubbers (Figure 1). The pyrolysis process was performed for approximately 3 h and the temperature was changed gradually to ensure steady conditions with minimal turbulence and a the similar state of the bed, hence the flow of the CO_2_ was decreased decreasing along with rising temperature. When the temperature had reached ca. 700 °C and no hydrocarbons had been emitted (Figure 2), the process was being continued for a further 30 min and then stopped by turning off the heating and increasing the CO_2_ flow. The mass of the obtained biochar was 38.1 g, so in the solid form retained ca. 19 wt.% of the SCG.

### 2.3. Adsorption Experiments

The processes of adsorption were carried out in 60 cm^3^ polypropylene flasks. Different masses of SCG and A-SCG, approximately 0.02, 0.05, 0.10, 0.20 and 0.50 g were added separately and mixed with 20 cm^3^ of metal ion aqueous solutions with the initial concentrations ranging from 100 mg/dm^3^ to 300 mg/dm^3^ of each metal ion. Probes were then shaken at room temperature for 24 h on the rotary shaker at 300 rpm. The temperature of the process was equal to 25 °C and the pH of the solutions was around 7, which is the pH of the used material. Preliminary studies showed that changing the pH did not substantially change the sorption properties of the material, thus the initial pH was selected. Additionally the process of adjusting the pH would not be beneficial as the material has buffering properties.

The obtained solution was filtrated through Pureland CA 0.45 µm filters and analyzed for the presence of metal ions such as Cd(II), Mn(II) and Pb(II), respectively. Each sorption process was triplicated and the obtained results were averaged. Sorption capacity at a given time (q_t_), at equilibrium (q_e_) and the percentage removal of metal ions (R_e_) was calculated using the equations showed below (Equations (1)–(3)) [21,22,23]:

(1) $$q_{e}=\frac{\left(C_{0}-C_{e}\right)\cdot V}{m\cdot 1000}$$

(2) $$q_{t}=\frac{(C_{0}-C_{t})\cdot V}{m\cdot 1000}$$

(3) $$R_{e}=\frac{C_{0}-C_{e}}{C_{0}}\cdot 100\%$$

### 2.4. Equilibrium Studies

Equilibrium parameters for Cd(II), Mn(II) and Pb(II) ions were calculated using the Langmuir, Freundlich and Temkin isotherms (Equations (4)–(7)) [24].

The Langmuir isotherm can be presented by the linearized equation presented below:

(4) $$\frac{C_{e}}{q_{e}}=\frac{C_{e}}{q_{\max}}+\frac{1}{K_{L}\cdot q_{\max}}$$

The Freundlich isotherm model is represented by Equation (5):

(5) $$\log q_{e}=\log K_{F}+\frac{1}{n}\log C_{e}$$

The Temkin isotherm has a linear form, as presented by Equations (6) and (7):

(6) $$q_{e}={\mathrm{BlnK}}_{T}+{\mathrm{BlnC}}_{e}$$

(7) $$B=\frac{\mathrm{RT}}{b_{t}}$$

### 2.5. Sorption Kinetics

Studies of the adsorption kinetics were carried out to determine the time required to reach the equilibrium of the process. The sorption capacities were measured at different times for different initial concentrations. The obtained results were used for the following kinetic models:

#### 2.5.1. Pseudo-First Order Rate Model

In the pseudo-first rate model, the linear equation for this model is represented by Equation (8) [25]:

(8) $$\ln\left(q_{e}-q_{t}\right)={\;\mathrm{lnq}}_{e}-k_{1}t$$

#### 2.5.2. Pseudo-Second Order Rate Model

The pseudo-second order model is represented by Equation (9):

(9) $$\frac{t}{q_{t}}=\frac{1}{k_{2}q_{e}^{2}}+\frac{t}{q_{e}}$$

#### 2.5.3. Intra Particle Diffusion Model

The Weber-Morris model is presented by the linear equation below:

(10) $$q_{t}=k_{\mathrm{id}}t^{0.5}+I$$

## 3. Results and Discussion

### 3.1. Characteristic of Sorbents

**Figure 3:**
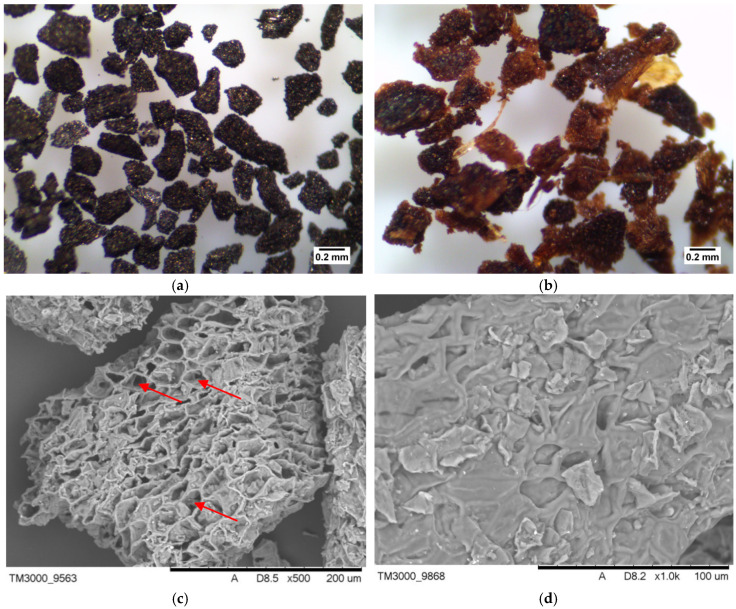
Optical (×40) and SEM microphotographs of (**a**) A-SCG, (**b**) SCG; (**c**) A-SCG, (**d**) SCG. Red arrows show the caverns (increasing the sorption capacity), which occurred after the fluidization process.

Figure 3 shows the optical microphotographs (×40 magnification) and SEM microphotographs along of A-SCG and SCG samples. The organic materials used in the study had a porous and rough surface. It can be seen that the spent coffee grounds had more porous and cellular-like structure after thermal process than the unmodified one.

According to the SEM-EDS analysis, the main elements of the tested materials were carbon and oxygen. Additionally the presence of potassium, silicone and phosphorus was found.

The C/H/N elemental analysis was determined and calculated based on the dry state of the samples. The results for A-SCG and SCG were C_%_ = 74.8; H_%_ = 1.6; N_%_ = 3.8; O_%_ = 19.8; and C_%_ = 9.3; H_%_ = 2.3; N_%_ = 0.8; O_%_ = 87.6, respectively. Pyrolysis led to significant carbonization of the material and is clearly evidenced by an increase in the mass fraction of coal and a decrease in the mass fraction of hydrogen in the sample.

**Figure 4:**
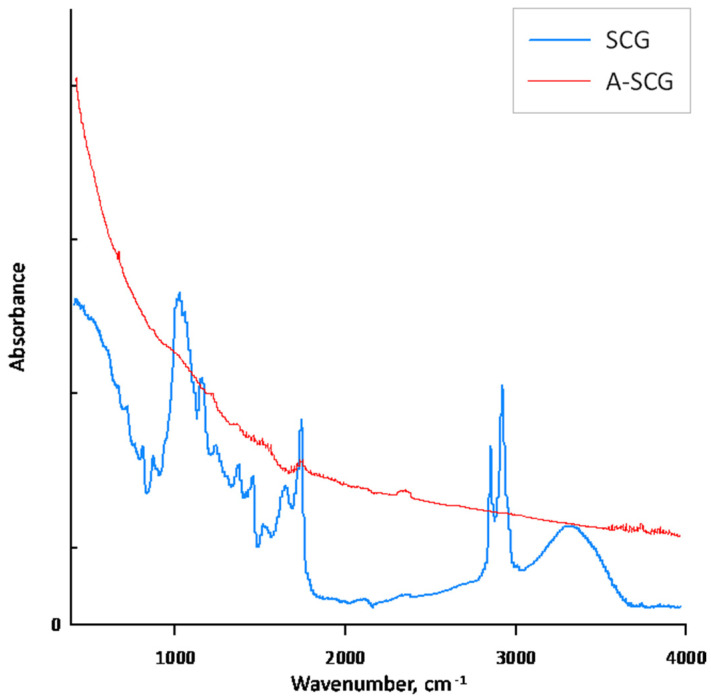
**Fourier Transform Infrared** (FTIR) spectra of used sorbent materials.

BET analysis showed that A-SCG had a surface area of 6.8 m^2^/g. Unfortunately, the surface analysis of coffee SCG was impossible to measure due to the degassing problems of the probe. According to Plaza et al. [15], high burn-off degrees of spent coffee grounds, which is equivalent to long residence time lead, to the widening of the micropores, thus obtaining a low value of surface area confirms this observation and points out that A-SCG is characterized by a microporous structure. Furthermore, Cho et al. [16] compared the pyrolysis of spent coffee grounds in both a CO_2_ and N_2_ environment and concluded that biochar from the CO_2_ environment exhibited higher porosity with a decreased number of active sites, and that this material would be favourable for retaining nutrients. The FTIR spectrum of the SCG exhibited the characteristic peaks of the cellulose structure, which were absent for A-SCG, (Figure 4). The broad peak at 3427 cm^−1^ is connected with the vibration of hydroxyl groups (OH^−^). The peak at 1032 cm^−1^ shows the presence of amino groups (NH_2_). The peak shown at 1622 cm^−1^ is consistent with the presence of carbonyl groups (COO^−^), and the last peak at 2920 cm^−1^ corresponds to the presence of the asymmetric stretch of CH_2_. In the spectrum of the A-SCG, one can see that most of the characteristic peaks were lowered due to the carbonization process. This can be compared to the FTIR spectra of active carbon or graphite from the literature data [25], which shows that the pyrolysis process effectively transformed coffee into biochar.

### 3.2. Adsorption of Cd(II), Pb(II) and Mn(II) Ions in a Batch System

**Figure 5:**
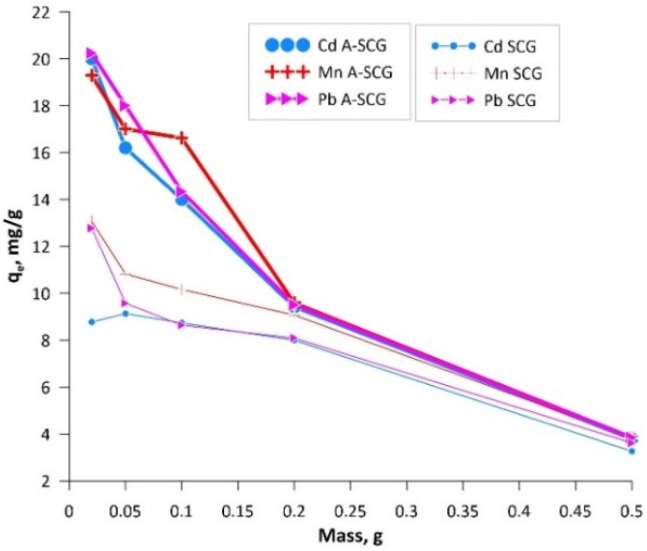
Graph of the adsorption of Cd(II), Mn(II) and Pb(II) ions at constant concentration versus mass of SCG and A-SCG.

The data presented in Figure 5 shows the adsorption degree of different metal ions under varying sorbent mass and constant ion concentration. Probes were shaken for 24 h to ensure sorption equilibrium. Additionally, in the figure shown below, one can see the sorption of Cd(II), Pb(II) and Mn(II) ions as a function of mass and adsorption. The changes depend on the amount and type of material used in the experiment. The differences can be explained due to the structural changes and development of the surface area between the SCG and A-SCG during the pyrolysis process. As shown in Figure 4, thermal activation caused the disappearance of IR radiation absorption for characteristic wavelengths related to the chemical composition of the untreated sorbent (saccharides, proteins, fats). This fact indicates the disappearance of functional groups, which are characterized by the presence of polar covalent bonds, which confirms the carbonization of activated material. The material is transformed in such a way that its structure begins to resemble the structure of graphite, which can be compared to the literature data [25]. Aromatization of the organic structure results in ring-delocalized orbitals (or in the form of condensed rings), which predisposes such material to form donor-acceptor bonds with metal cations. These bonds are π_d_cation-p_del_C_ type bonds, where electron pair acceptors are metal cations and electron pairs come from the sorbent surface. No such bonds occur without sorbent aromatization.

### 3.3. Equilibrium Analysis

**Figure 6:**
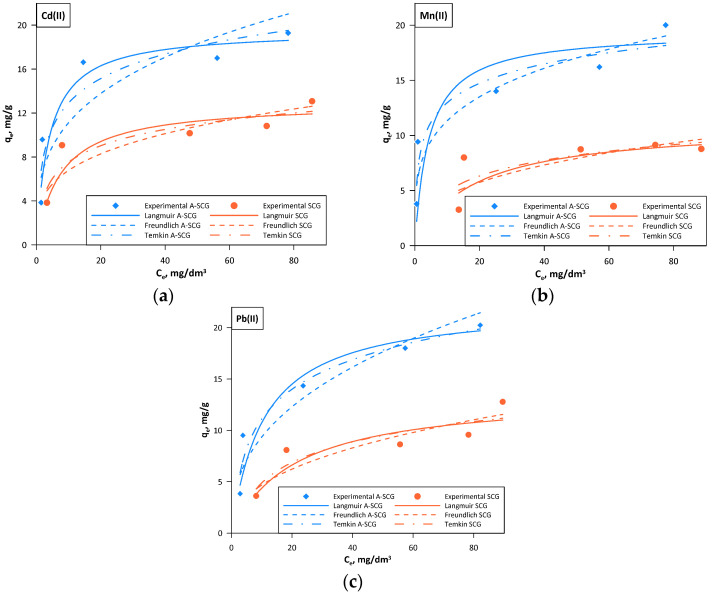
Langmuir, Freundlich and Temkin isotherms for A-SCG and SCG. (**a**) Cadmium; (**b**) Manganese; (**c**) Lead.

Sorption capacity of the used materials was determined by using the Langmuir, Freundlich and Temkin isotherms. Based on the experimental data, it can be concluded that with increasing mass ratio of adsorbent to concentration of metal ions (Cd(II), Mn(II), Pb(II)), the sorption capacity decreased from 20.24 mg/g to 3.83 mg/g for A-SCG and from 12.78 mg/g to 3.62 mg/g for SCG. In addition, it can be seen that the materials could remove between 9 to 99% of ions depending on the initial mass of the materials and the ion used. Figure 6 shows the linearized forms of the studied isotherms. On their basis, the parameters of the isotherms were calculated and are summarized in Table 1.

Based on the obtained results one can see that the best model describing the sorption of the used metal ions on both A-SCG and SCG was the Langmuir isotherm model, which had the highest correlation coefficient R^2^ in all of the tested sets.

Constant R_L_ is connected with the nature of sorption. Equation (11) was used to calculate the constant R_L_:

(11) $$R_{L}=\frac{1}{1+K_{F}C_{0}}$$

For R_L_ parameters:

R_L_ = 0—sorption is irreversible,

0 < R_L_ < 1—favorable sorption conditions,

R_L_ = 1—linear nature of the sorption,

R_L_ > 1—unfavorable conditions for sorption.

The obtained data showed that the constant R_L_ was always positive and lower than 1, which indicates that the process of adsorption of the examined metal ions is favorable.

It can be concluded that the process of sorption of Pb(II) ions, Cd(II) ions and Mn(II) ions onto the spent coffee grounds is of a chemical nature, where the rate-limiting step is the rate of surface reactions.

Maximum sorption capacity for A-SCG was equal to 22.3 mg/g, 19.6 mg/g and 19.4 mg/g for Pb(II) ions, Cd(II) ions and Mn(II) ions, respectively. These values where respectively 64%, 51% and 77% higher for A-SCG than for SCG.

### 3.4. Kinetic Studies

**Figure 7:**
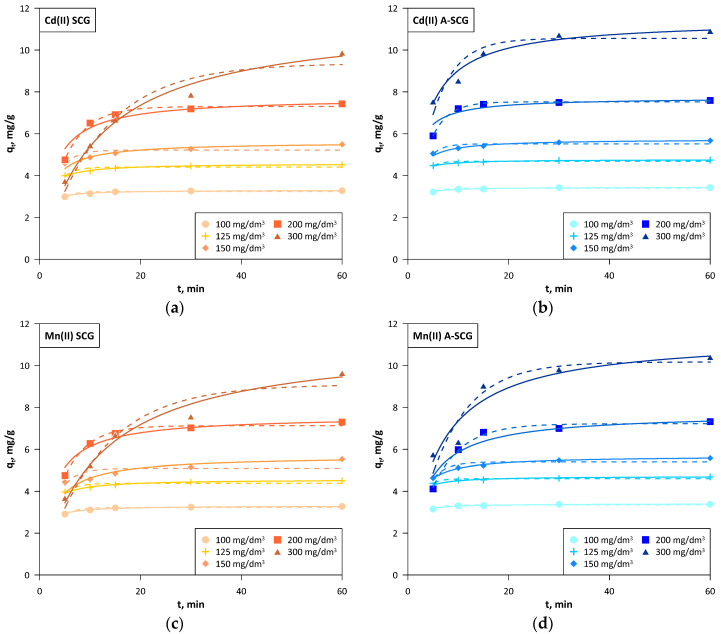

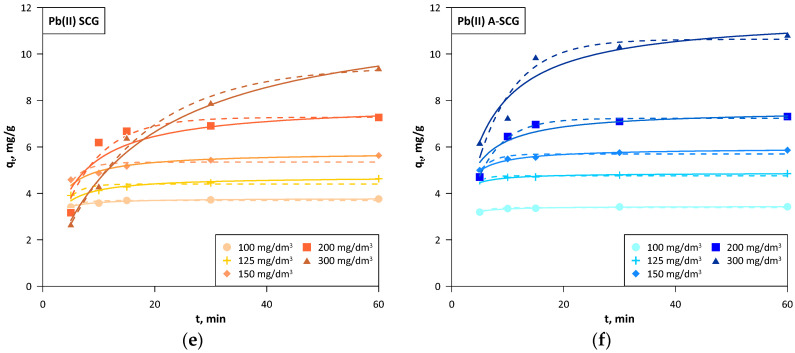
Kinetics of the linear pseudo-second order (solid lines) and non-linear pseudo-first order (dashed lines) models for A-SCG and SCG. (**a**) Cadmium, SCG; (**b**) Cadmium, A-SCG; (**c**) Manganese, SCG; (**d**) Manganese, A-SCG; (**e**) Lead, SCG; (**f**) Lead, A-SCG.

In order to find the physicochemical characteristics of the mechanism’s process of sorption, the kinetics studies were performed. It seems that there are a couple steps in the adsorption process. The first stage is connected with the diffusion of metal ions on the surface of the sorbent and further into the pores. The next stage starts with the metal interaction with the sorbent active sites. In order to compare different kinetics models the correlation coefficient (R^2^) was calculated. Three kinetic models were used such as the pseudo-first, pseudo-second and Webber-Morris model (Figure 7 and Table 2, Table 3 and Table 4). The solid lines show the second order linear fit and the dashed lines show the first order non-linear fit, which were the best fits to explain the adsorption of metal ions onto the SCG and A-SCG. In addition, the Chi-square test [26] was used to better compare the suitability of the model to the experimental results:

(12) $$\chi^{2}=\frac{{\left(q_{e\;\exp}-q_{e\;\mathrm{cal}}\right)}^{2}}{q_{e\;\mathrm{cal}}}$$

The last experimental point in each case was taken into account for calculating q_e exp_.

The linear pseudo first order model due to the high error in the χ^2^ and no adjustment to the experimental data was not the best fit for the described study. The non-linear pseudo first order model showed low error in the χ^2^, but low correlation coefficient R^2^. In addition, it assumed lower values at the last point (60 min) below the experimental data in each case, which can be clearly seen in the graphs, which might suggest that equilibrium was reached earlier. The non-linear pseudo second order model generally provides a larger χ^2^ error than the linear models and a lower correlation coefficient R^2^, but the obtained values and the presented graph were very close to that of the linear model [27]. The linear pseudo-second order had a slightly higher χ^2^ error than that in a linear method, but had a very high correlation coefficient R^2^ with satisfactory compliance with the experimental data. In this model, the values of q_e cal_ were in some cases higher than the last experimental point on the graph, mainly seen in A-SCG at initial concentrations over 150 mg/dm^3^. This suggests that the time of the experiment is not always sufficient to reach the equilibrium, but the extrapolation of the linear second-order plot can supply respective values.

Based on the kinetic data, it can be observed that for both the A-SCG and SCG and for all of the tested metal ions, the model with the best fit was the linear form of the pseudo-second order. The sorption equilibrium set in the early stage of the sorption process (on average 30 min for concentrations below 200 mg/dm^3^) for both used materials and all of the metal ions. The average sorption rate in the first 5 min at an initial concentration of 300 mg/dm^3^ was 1.51, 1.15 and 1.24 mg/g/min for A-SCG, and this value was higher than 2.0, 1.6 and 2.3 times than for SCG for Cd(II), Mn(II) and Pb(II), respectively. Based on this information, it can be assumed that the process that limits the sorption of used metal ions is chemisorption, which is based on the influence of valence bonds through the sharing or exchange of electrons between ions and the sorbent.

## 4. Conclusions

The research showed that activated spent coffee grounds during the pyrolysis process in the fluidized bed reactor undergo substantial physical modification at the macroscopic and microscopic level. Thermal activation of SCG in the fluidized bed reactor not only improved sorption, but also provides a safe and stable sorbent that does not release organic substances into water solutions and can be easily separated from the mixture. A-SCG has the form of a porous biochar that can be used for the effective and rapid removal of metal ions from aqueous solutions.

The best model fit for the studied process was the Langmuir isotherm, and the pseudo-second order kinetic model correctly described the kinetics process. Thus, it can be concluded that the process of sorption of Pb(II) ions, Cd(II) ions and Mn(II) ions onto spent coffee grounds is of a chemical nature, where the rate-limiting step is the rate of the surface reactions.

Maximum sorption capacity derived from the Langmuir model for A-SCG was equal to 22.3 mg/g, 19.6 mg/g and 19.4 mg/g for the Pb(II) ions, Cd(II) ions and Mn(II) ions, respectively. Furthermore, these values were respectively 64%, 51% and 77% higher for A-SCG than for SCG.

The sorption equilibrium set in the early stage of the sorption process (on average 30 min for concentrations below 200 mg/dm^3^) for both of the used materials and all of the metal ions. Kinetic models were calculated for both linear and non-linear forms and by further introduction of an additional statistical test, it was possible to obtain confirmation of reasonable fit with the experimental data.

## Figures and Tables

**Table 1:** Isotherm parameters.

	Cd(II)		Mn(II)		Pb(II)	
	A-SCG	SCG	A-SCG	SCG	A-SCG	SCG
**Langmuir**						
**K_L_, dm^3^/mg**	0.2495	0.1318	0.2289	0.0566	0.0937	0.0477
**q_max_, mg/g**	19.56	12.97	19.40	10.99	22.25	13.58
**R_L_**	0.0392	0.0718	0.0435	0.1553	0.0961	0.1726
**R^2^**	0.9921	0.9714	0.9717	0.9200	0.9877	0.9008
**Freundlich**						
**K_F_**	5.448	3.483	6.329	2.015	3.785	1.817
**1/n**	0.3097	0.2891	0.2529	0.3494	0.3935	0.4116
**R^2^**	0.7419	0.7778	0.8151	0.5004	0.8174	0.8227
**Temkin**						
**K_T_**	5.601	3.326	16.717	1.091	1.462	0.552
**B**	3.209	2.150	2.534	2.050	4.151	2.867
**R^2^**	0.8695	0.8290	0.8970	0.5524	0.9391	0.8137

**Table 2:** Values of the parameters characterizing the Cd(II) sorption kinetics.

C_o_, mg/dm^3^	q_e exp_, mg/g	Pseudo-First Order (Non-linear)				Pseudo-Second Order (Non-linear)		Pseudo-Second Order (Linear)				Webber-Morris		
		q_e cal_, mg/g	k_1_, 1/min	χ^2^	R^2^	χ^2^	R^2^	q_e cal_, mg/g	k_2_, mg/g/min	χ^2^	R^2^	I	k_id_, mg/g·min^1/2^	R^2^
**Cd(II) SCG**														
**100**	3.28	3.24	0.504	0.001	0.846	0.001	0.984	3.31	0.648	0.000	1.000	2.98	0.046	0.684
**125**	4.52	4.41	0.456	0.003	0.784	0.000	0.994	4.58	0.275	0.001	1.000	3.92	0.088	0.807
**150**	5.49	5.22	0.398	0.014	0.665	0.000	0.948	5.61	0.121	0.002	1.000	4.36	0.156	0.928
**200**	7.43	7.30	0.212	0.002	0.991	0.036	0.938	7.74	0.056	0.012	0.999	4.79	0.393	0.638
**300**	9.87	9.36	0.085	0.027	0.946	0.181	0.985	11.56	0.008	0.247	0.995	1.98	1.054	0.962
**Cd(II) A-SCG**														
**100**	3.43	3.40	0.589	0.000	0.832	0.000	0.984	3.45	0.883	0.000	1.000	3.21	0.033	0.715
**125**	4.74	4.69	0.610	0.001	0.768	0.000	0.989	4.77	0.585	0.000	1.000	4.44	0.045	0.790
**150**	5.67	5.52	0.482	0.004	0.726	0.000	0.981	5.75	0.216	0.001	1.000	4.94	0.104	0.862
**200**	7.59	7.53	0.307	0.000	0.996	0.016	0.901	7.73	0.129	0.003	1.000	6.07	0.232	0.525
**300**	10.91	10.56	0.213	0.011	0.857	0.033	0.963	11.45	0.033	0.025	0.999	6.81	0.600	0.805

**Table 3:** Values of the parameters characterizing the Mn(II) sorption kinetics.

C_o_, mg/dm^3^	q_e exp_, mg/g	Pseudo-First Order (Non-linear)				Pseudo-Second Order (Non-linear)		Pseudo-Second Order (Linear)				Webber-Morris		
		q_e cal_, mg/g	k_1_, 1/min	χ^2^	R^2^	χ^2^	R^2^	q_e cal_, mg/g	k_2_, mg/g/min	χ^2^	R^2^	I	k_id_, mg/g·min^1/2^	R^2^
**Mn(II) SCG**														
**100**	3.28	3.22	0.457	0.001	0.886	0.001	0.986	3.31	0.488	0.000	1.000	2.90	0.056	0.688
**125**	4.51	4.38	0.453	0.004	0.753	0.000	0.988	4.57	0.251	0.001	1.000	3.87	0.092	0.840
**150**	5.53	5.09	0.373	0.038	0.456	0.002	0.819	5.70	0.076	0.005	0.999	4.00	0.202	0.985
**200**	7.30	7.13	0.215	0.004	0.987	0.028	0.961	7.60	0.055	0.012	1.000	4.69	0.384	0.683
**300**	9.64	9.09	0.087	0.033	0.935	0.159	0.976	11.25	0.008	0.230	0.992	1.97	1.021	0.953
**Mn(II) A-SCG**														
**100**	3.39	3.35	0.560	0.000	0.889	0.000	0.979	3.41	0.827	0.000	1.000	3.15	0.036	0.673
**125**	4.69	4.60	0.604	0.002	0.712	0.000	0.956	4.72	0.432	0.000	1.000	4.33	0.050	0.873
**150**	5.58	5.40	0.376	0.006	0.839	0.001	0.995	5.69	0.149	0.002	1.000	4.52	0.153	0.802
**200**	7.32	7.22	0.173	0.001	0.990	0.063	0.932	7.74	0.040	0.023	0.999	4.08	0.481	0.654
**300**	10.40	10.17	0.130	0.005	0.882	0.113	0.908	11.42	0.016	0.091	0.995	4.39	0.863	0.797

**Table 4:** Values of the parameters characterizing the Pb(II) sorption kinetics.

C_o_, mg/dm^3^	q_e exp_, mg/g	Pseudo-First Order (Non-linear)				Pseudo-Second Order (Non-linear)		Pseudo-Second Order (Linear)				Webber-Morris		
		q_e cal_, mg/g	k_1_, 1/min	χ^2^	R^2^	χ^2^	R^2^	q_e cal_, mg/g	k_2_, mg/g/min	χ^2^	R^2^	I	k_id_, mg/g·min^1/2^	R^2^
**Pb(II) SCG**														
**100**	3.76	3.70	0.500	0.001	0.833	0.000	0.981	3.79	0.499	0.000	1.000	3.39	0.055	0.708
**125**	4.63	4.40	0.416	0.012	0.634	0.000	0.936	4.72	0.149	0.002	1.000	3.72	0.125	0.941
**150**	5.63	5.35	0.362	0.015	0.667	0.000	0.947	5.77	0.104	0.004	1.000	4.31	0.184	0.910
**200**	7.27	7.28	0.148	0.000	0.923	0.121	0.839	7.87	0.029	0.046	0.992	3.47	0.572	0.561
**300**	9.40	9.48	0.066	0.001	0.988	0.575	0.983	12.05	0.005	0.583	0.993	0.77	1.193	0.918
**Pb(II) A-SCG**														
**100**	3.43	3.39	0.562	0.000	0.889	0.000	0.979	3.45	0.827	0.000	1.000	3.19	0.036	0.673
**125**	4.85	4.76	0.612	0.002	0.713	0.000	0.956	4.88	0.437	0.000	1.000	4.49	0.049	0.871
**150**	5.86	5.70	0.408	0.005	0.856	0.001	0.990	5.95	0.172	0.001	1.000	4.92	0.136	0.776
**200**	7.31	7.23	0.214	0.001	0.994	0.040	0.917	7.59	0.062	0.010	0.999	4.81	0.376	0.595
**300**	10.84	10.64	0.147	0.004	0.909	0.102	0.917	11.70	0.019	0.063	0.997	5.21	0.820	0.752

## Symbols

C_o_	initial concentration of metal ions (mg/dm^3^)
C_e_	concentration of metal ions at equilibrium (mg/dm^3^)
q_e_	sorption capacity at equilibrium (mg/g)
q_t_	sorption capacity at any time (mg/g)
R_e_	the percentage removal at equilibrium
q_max_	maximum sorption capacity (mg/g)
K_L_	Langmuir constant (L/mg)
K_F_	Freundlich constant (mg^1−(1/n)^(dm^3^)^1/n^g^−1^)
n	heterogeneity factor
K_T_	the equilibrium binding constant corresponding to the maximum binding energy (dm^3^/g)
B	constant related to the heat of sorption (J/mol)
b_t_	Temkin isotherm constant
k_1_	the pseudo-first order rate constant (1/min)
k_2_	the pseudo-second order rate constant (g/(mg·min))
k_id_	the intra-particle diffusion rate constant (mg/g·min^0.5^)
I	intercept of the line in Weber-Morris model
q_e exp_	experimental amount of metal adsorbed at equilibrium (mg/g)
q_e cal_	calculated amount of metal adsorbed at equilibrium (mg/g)
